# Pharmacological inhibition of EZH2 disrupts the female germline epigenome

**DOI:** 10.1186/s13148-018-0465-4

**Published:** 2018-03-05

**Authors:** Lexie Prokopuk, Kirsten Hogg, Patrick S. Western

**Affiliations:** 1 Centre for Reproductive Health, Hudson Institute of Medical Research, Clayton, Victoria 3168 Australia; 2 Department of Molecular and Translational Science, Monash University, Clayton, Victoria 3168 Australia

**Keywords:** Germline, Oocyte, Pharmacology, Epigenetic, PRC2, H3K27me3, Inheritance

## Abstract

**Background:**

Recently discovered drugs that target epigenetic modifying complexes are providing new treatment options for a range of cancers that affect patients of reproductive age. Although these drugs provide new therapies, it is likely that they will also affect epigenetic programming in sperm and oocytes. A promising target is Enhancer of Zeste 2 (EZH2), which establishes the essential epigenetic modification, H3K27me3, during development.

**Results:**

In this study, we demonstrate that inhibition of EZH1/2 with the clinically relevant drug, tazemetostat, severely depletes H3K27me3 in growing oocytes of adult female mice. Moreover, EZH2 inhibition depleted H3K27me3 in primary oocytes and in fetal oocytes undergoing epigenetic reprogramming. Surprisingly, once depleted, H3K27me3 failed to recover in growing oocytes or in fetal oocytes.

**Conclusion:**

Together, these data demonstrate that drugs targeting EZH2 significantly affect the germline epigenome and, based on genetic models with oocyte-specific loss of EZH2 function, are likely to affect outcomes in offspring.

**Electronic supplementary material:**

The online version of this article (10.1186/s13148-018-0465-4) contains supplementary material, which is available to authorized users.

## Background

Although many studies indicate that epigenetic changes in gametes can influence development and health in offspring, the mechanisms are poorly understood (reviewed in [1, 2]). Despite this, new pharmaceuticals that specifically target epigenetic modifying enzymes are currently being developed for the treatment of cancer and other diseases, and these drugs are being administered to patients of reproductive age. While these therapies are likely to offer significant improvements in cancer treatment, their potential impacts on the germline epigenome, and on offspring, remain unknown.

Altered epigenetic states can result in disease, and more than 50% of cancers harbour mutations in chromatin-associated proteins. This has driven recent interest in the development of drugs that specifically target epigenetic modifying complexes with the aim of providing new cancer treatments [3]. A prominent target is Enhancer of Zeste 2 (EZH2), which is commonly subject to gain of function mutations or overexpression in a range of tumours [3–6]. EZH2 catalyses methylation of lysine 27 on histone 3 (H3K27me3), an epigenetic modification that is critical for repressing developmental genes during embryogenesis. EZH2 is the catalytic component of polycomb repressive complex 2 (PRC2), which also contains embryonic ectoderm development (EED) and Suppressor of Zeste 12 (SUZ12). Inactivation of any one of these three protein subunits severely compromises the enzymatic activity of PRC2 and results in the loss of H3K27me3 [7–10].

Recently developed pharmaceuticals that inhibit EZH2 include EPZ-6438, also known as the proprietary product, tazemetostat [11]. Tazemetostat is currently in stage I/II clinical trials in patients for the treatment of multiple cancers, including lymphomas, mesothelioma, myelomas, solid tumours, and malignant rhabdoid tumours of the kidney and ovary. Patient cohorts include children and adults of reproductive age (12 current NIH Clinical Trials 02860286, 02875548, 03028103, 03010982, 02601937, 01897571, 02601950, 03009344, 03213665, 02220842, 03217253, 03155620). Three studies include the recruitment of children, aged 6 months to 21 years (NIH Clinical Trials 02601937, 03213665, 03155620).

As H3K27me3 is enriched in developing germ cells during epigenetic reprogramming and in mature oocytes [12–16], it is likely that drugs targeting EZH2 will disrupt the sperm and oocyte epigenome. Current guidelines for tazemetostat typically recommend avoiding pregnancy for 30 days post-treatment (NIH Clinical Trials Register), significantly less time than the ~ 350 days required for human oocyte growth and maturation [17]. Other studies do not stipulate any form of contraception after treatment with tazemetostat. As these drugs are systemic and the germline will be exposed in patients undergoing treatment, determining whether current treatments affect the germline epigenome is of significant importance for developing informed therapeutic guidelines and providing advice to patients with regard to post-treatment reproductive management. Moreover, although the use of these drugs during pregnancy is contra-indicated, there is little empirical data to support this advice. This is important, as recent studies have provided examples of drugs taken during pregnancy that have disrupted fetal or ovarian development [18, 19]. Of particular note, the epigenetic modifying drug valproic acid (Epilim) has been extensively used for the treatment of epilepsy, bipolar mania and migraine prophylaxis, but recent evidence of affects in children of women who took the drug during pregnancy have raised concerns over its clinical management [19]. While the effects of these, and other drugs, on the fetus have been of central concern, their potential impacts on the germline epigenome have been largely overlooked. These examples underline the need to understand the germline effects of epigenetic modifying drugs to allow appropriate development of informed clinical guidelines.

The developing germline plays a central role in regulating epigenetic information transmitted by gametes to offspring. To ensure transmission of appropriate epigenetic information to offspring, epigenetic information is reset during germline development [14, 20–24]. During this period of germline epigenetic reprogramming, H3K27me3 is highly enriched in primordial germ cells and in both male and female germ cells compared to somatic cells of the developing gonad [13, 14, 21, 25]. Moreover, H3K27me3 undergoes EZH2-dependent reorganisation while DNA methylation levels are at their lowest, indicating important roles for EZH2 and H3K27me3 for setting the germline epigenome [14]. Consistent with this, genome-wide mapping demonstrated H3K27me3 enrichment at developmental genes during epigenetic programming in the mouse fetal germline and in mature sperm [15, 26–29]. Given that many of these genes are not expressed during germline development, it has been speculated that these epigenetic modifications are established to regulate outcomes in offspring [15]. This concept is consistent with observations that deletion of *Ezh2* in growing oocytes affects preimplantation development and birth weight in offspring [16].

Females are born with a finite pool of primordial follicles, which provide the lifelong oocyte reserve that supports fertility. Primordial follicles are continually activated during reproductive life, resulting in the formation of primary follicles and oocyte growth [30–32]. As activated follicles grow from primary to pre-ovulatory stages, epigenetic modifications and maternal factors are established in the oocyte that are important for offspring development [33]. These maternal factors are produced through co-ordinate regulation of gene expression in the growing oocyte, a process that is considered to depend on appropriate epigenetic regulation throughout this period. An important example is provided by EZH2, which performs an essential role in the oocyte that profoundly affects offspring development and growth [16].

Here, we show that the EZH1/2-specific inhibitor, tazemetostat, substantially alters H3K27me3 in the developing female germline and maturing oocytes. Tazemetostat severely reduced H3K27me3 in growing oocytes in vivo, and H3K27me3 was not recovered after treatment withdrawal for half of the mouse oocyte growth period. Moreover, tazemetostat significantly reduced H3K27me3 in developing germ cells and in primary oocytes. Together, these data demonstrate that systemic pharmacological inhibition of EZH2 affects H3K27me3 at all stages of oocyte development and growth. While tazemetostat and similar drugs that target epigenetic pathways offer important therapeutic options to patients, our findings highlight an urgent need for a greater understanding of their impacts on the germline and the reassessment of clinical guidelines to ensure safe management in patients who may wish to conceive after treatment.

## Results

### PRC2 and H3K27me3 are enriched in growing oocytes of adult female mice

**Fig. 1:**
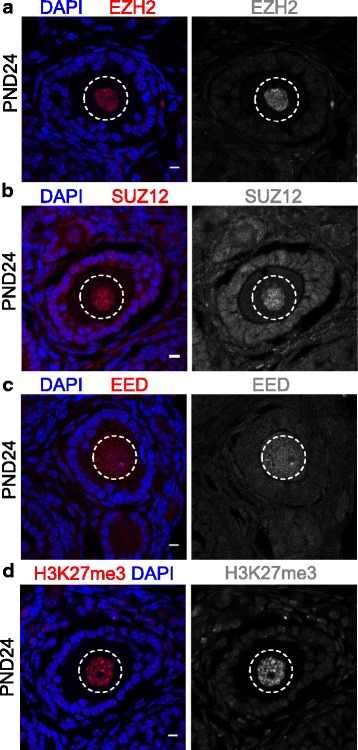
PRC2 and H3K27me3 are enriched in growing oocytes. Confocal images of EZH2, SUZ12 and EED immunofluorescence in sections of ovary at postnatal day 24. Left panels show merged images. **a** EZH2 (red) and DAPI (DNA; blue). **b** SUZ12 (red) and DAPI (DNA; blue). **c** EED (red) and DAPI (DNA; blue). **d** H3K27me3 (red) and DAPI (DNA; blue). Right panels show single channel images of EED/EZH2/SUZ12/H3K27me3 (greyscale). Oocyte nuclei are shown within the white dashed line. Images are representative of three biological replicates. 10 μm scale bars

To determine the potential for PRC2 to catalyse H3K27me3 in growing oocytes of adult females, we assessed EZH2, EED, SUZ12 and H3K27me3 enrichment in postnatal day (PND) 24 ovaries using immunofluorescent staining. EED, EZH2 and SUZ12 were all detected in the nucleus of growing oocytes in PND24 ovaries (Fig. 1a–c), coinciding with H3K27me3 enrichment (Fig. 1d).

### EZH2 inhibition potently depletes H3K27me3 in growing oocytes

**Fig. 2:**
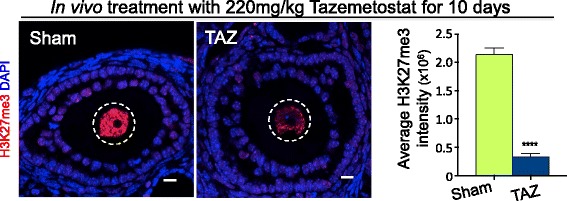
Tazemetostat treatment of adult female mice substantially reduced H3K27me3 in growing oocytes. Confocal images of immunofluorescence in ovary sections from adult mice treated for 10 days with vehicle control (sham; 0.5% NaCMC, 0.1% Tween 80) or 220 mg/kg tazemetostat injected daily. Each panel shows merged colour images with H3K27me3 shown in red and DAPI (DNA) in blue. Oocyte nuclei are shown within the white dashed line. Images are representative of three biological replicates. 10 μm scale bars. Mean fluorescence intensity for H3K27me3 is shown in the graph to the right analysed by ROI manager, ImageJ. ****P < 0.0001 one-way ANOVA plus post hoc Tukey’s multiple comparisons test; *n* = 4 biological replicates per treatment, 5–7 oocytes measured per replicate. Error bars ± SEM

As tazemetostat and other similar drugs are systemic, treatment of females of reproductive age is expected to result in oocyte exposure to these EZH2-inhibiting drugs. To determine whether pharmacological inhibition of EZH1/2 affected H3K27me3 enrichment in the adult female germline, we treated adult females with tazemetostat. This is an especially important question as females are born with a finite supply of oocytes and disruption of oocyte epigenetic state may affect offspring outcomes. Previous in vivo preclinical studies of tazemetostat have shown that 220 mg/kg administered twice daily is required to induce effective tumour regression in mouse models carrying grafted malignant rhabdoid cells [4]. To investigate whether this dose caused depletion of H3K27me3 in growing oocytes in vivo, 7-week-old adult female mice were injected subcutaneously with either 220 mg/kg tazemetostat (*n* = 3) or sham control (0.5% NaCMC, 0.1% Tween80; *n* = 3) for 10 days. Notably, we injected mice once a day, thereby delivering less drug than in the preclinical cancer model, in which the drug was administered twice daily at 220 mg/kg by oral gavage [4]. Immunofluorescent analysis of H3K27me3 enrichment in secondary follicles revealed that H3K27me3 was depleted by 84% in the oocytes of females injected with tazemetostat compared to sham injected controls (*P* < 0.0001, Student’s *t* test; Fig. 2). This demonstrated potent disruption of EZH1/2 function in growing oocytes of adult females.

### Adult oocytes failed to recover H2K27me3 after withdrawal of EZH2 inhibition

**Fig. 3:**
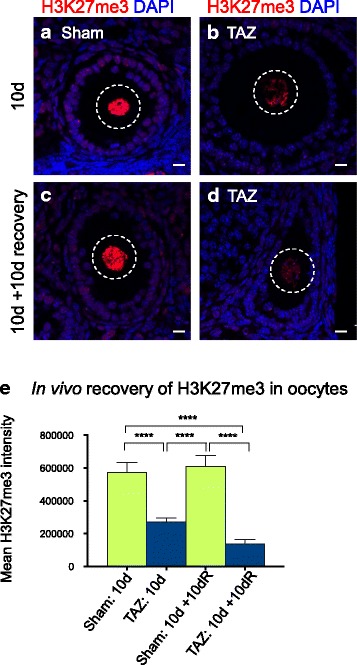
H3K27me3 was not recovered in oocytes of adult mice after tazemetostat treatment. Confocal images of immunofluorescence in ovary sections from adult mice treated. **a** Treatment for 10 days with vehicle control (sham; 0.5% NaCMC, 0.1% Tween 80). **b** Treatment for 10 days with 220 mg/kg tazemetostat injected daily. **c.** Treatment for 10 days with vehicle control (sham; 0.5% NaCMC, 0.1% Tween 80) followed by a 10-day no treatment recovery period. **d.** Treatment for 10 days with 220 mg/kg tazemetostat followed by a 10-day no treatment recovery period. Each panel shows merged colour images with H3K27me3 shown in red and DAPI (DNA) in blue. Oocyte nuclei are shown within the white dashed line. Images are representative of three biological replicates. 10 μm scale bars. **e.** Mean fluorescence intensity for H3K27me3 for each treatment, analysed by ROI manager, ImageJ. ****P < 0.0001 one-way ANOVA plus post hoc Tukey’s multiple comparisons test; *n* = 4 biological replicates, 7–12 oocytes measured per replicate. Error bars ± SEM

To assess whether growing oocytes could recover after the extensive loss of H3K27me3 resulting from tazemetostat treatment, we carried out an in vivo recovery experiment. One cohort of 7-week-old adult female mice were injected subcutaneously with either 220 mg/kg tazemetostat (*n* = 3) or sham control (*n* = 3) once a day for 10 days and collected. An additional cohort was injected with either 220 mg/kg tazemetostat (*n* = 3) or sham control (*n* = 3) once a day for 10 days and then allowed to recover for a further 10 days with no treatment. After treatment or treatment/recovery regimes, immunofluorescence was performed on ovaries and secondary follicles to assess H3K27me3 enrichment in oocytes. Consistent with the initial in vivo depletion experiment in growing oocytes, H3K27me3 was robustly depleted in growing oocytes after 10 days of EZH2 inhibition compared to sham injected controls (Fig. 3). However, remarkably, we did not observe any H3K27me3 rescue in oocytes allowed to recover for 10 days (*P* < 0.0001, one-way ANOVA with post hoc Tukey’s multiple comparisons test; Fig. 3). Instead, after the 10-day recovery period, H3K27me3 was depleted by a further 50% in oocytes.

Together, these data demonstrate that systemic tazemetostat treatment at the minimal preclinical dose that effectively controls tumour growth in mice also results in severe depletion of H3K27me3 in maturing oocytes. Moreover, our data indicate that once depleted, H3K27me3 does not recover in a window corresponding to half the growth period for mouse oocytes.

### EZH2 inhibition depletes H3K27me3 in female fetal germ cells undergoing epigenetic reprogramming

Although use of tazemetostat during pregnancy is contra-indicated, empirical evidence for this position is lacking. Previous studies provide examples where similar drugs have been used in pregnancy [19], raising the possibility that drugs targeting epigenetic modifying enzymes may be similarly used in the future. Since exposure of the fetus would also expose the germline of future offspring to the drug, we determined the ability of EZH2 inhibitors to alter H3K27me3 in developing oocytes. To achieve this, we performed a series of experiments on germ cells using ex vivo cultured fetal gonads treated with tazemetostat. Germ cells undergo significant epigenetic reprogramming during fetal life that results in removal of existing epigenetic modifications and the establishment of new sex-specific germline epigenomes. This developmental period in germ cells is therefore considered susceptible to modification by agents that disrupt epigenetic programming and may alter the germline epigenome in mature gametes. As H3K27me3 is significantly reorganised in germ cells undergoing epigenetic reprogramming [14], we initially determined the potential for EZH2 inhibition to affect H3K27me3 enrichment in fetal germ cells during this period.

**Fig. 4:**
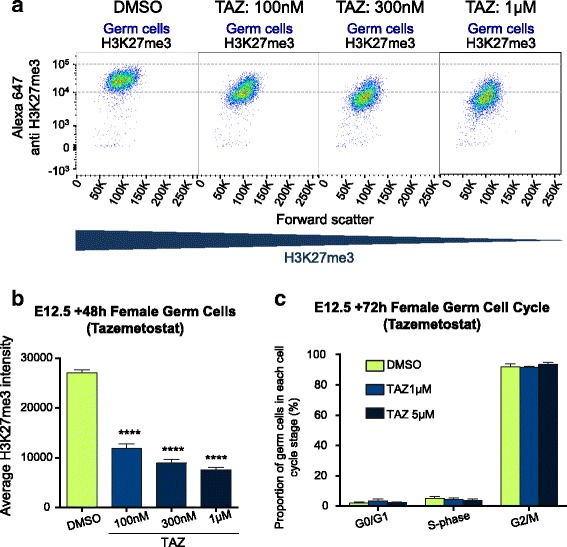
EZH2 inhibition potently reduced H3K27me3 levels in fetal oocytes undergoing epigenetic reprogramming. **a.** Flow scatter plots showing H3K27me3 mean intensity of germ cells measured by flow cytometry of XX E12.5 gonads cultured for 48 h with DMSO, 100 nM, 300 nM or 1 μM tazemetostat. Examples of flow cytometric gating are shown in Additional file 1: Figure S1. **b** Average H3K27me3 staining intensity in germ cells from XX female E12.5 gonads cultured for 48 h with DMSO, 100 nM, 300 nM, 1 μM tazemetostat**.** ****P < 0.0001, one-way ANOVA plus post hoc Tukey’s multiple comparisons test; *n* = 3–5 biological replicates. Error bars ± SEM. **c** Cell cycle state in germ cells from XX female E12.5 gonads cultured for 72 h with DMSO, 1 μM and 5 μM tazemetostat**.** No significant differences between DMSO control and treatments. One-way ANOVA plus post hoc Tukey’s multiple comparisons test; *n* = 3–5 biological replicates. Error bars ± SEM

Female Swiss mice were time-mated to *Oct4GFP* transgenic males (129T2svJ) to allow stage-specific collection of embryonic gonads with *Oct4GFP* expressed in germ cells but not in somatic cells [14, 34, 35]. We treated E12.5 XX (female) fetal gonads grown in ex vivo organ culture with tazemetostat or vehicle control (DMSO) for 24–72 h. At the end of the culture period, the gonads were dissociated and H3K27me3 levels measured in individual germ and somatic cells using flow cytometry ([14, 36]; Additional file 1: Figure S1). Robust depletion of H3K27me3 was observed in germ cells of XX E12.5 gonads treated for 72 h with 100 nM–5 μM tazemetostat (Fig. 4a, b; Additional file 1: Figure S1C). Treatment with 100 nM tazemetostat resulted in significant depletion of H3K27me3, although slightly less efficiently than that at higher doses. Tazemetostat was very well tolerated in culture, with even the highest doses having no noticeable effect on gonad growth or the proportions of germ cells at the end of the culture period (Fig. 4c, Additional file 1: Figures. S1C, S2A-B). This is consistent with our previous observations that cell viability was unaffected by a similar EZH2-inhibiting drug, GSK126, in this culture system [14].

A major milestone for female germ cell development is their entry into meiosis, which occurs between E13.5 and E15.5 [37, 38]. To assess the impact of EZH2 inhibitors on female germ cell development, E12.5 gonads were treated for 72 h allowing sufficient time for germ cells to enter meiosis. Cell cycle state was assessed by incorporation of EdU during the final 2 h of gonad culture and staining germ cells with propidium iodide to determine individual cell DNA content using flow cytometry ([38–40]; Additional file 1: Figure S2). XX germ cells arrested in meiosis with normal timing and in normal numbers in E12.5 gonads treated for 72 h with tazemetostat, indicating that EZH2 inhibition and loss of H3K27me3 did not prevent E12.5 germ cells from reaching this key developmental milestone (Fig. 4c). Similarly, somatic cells proliferated at a normal rate in XX gonads indicating that EZH2 inhibition did not impact gonad growth during the treatment period, despite loss of H3K27me3 (Additional file 1: Figure S2B).

### H3K27me3 failed to recover in female fetal germ cells following EZH2 inhibition

**Fig. 5:**
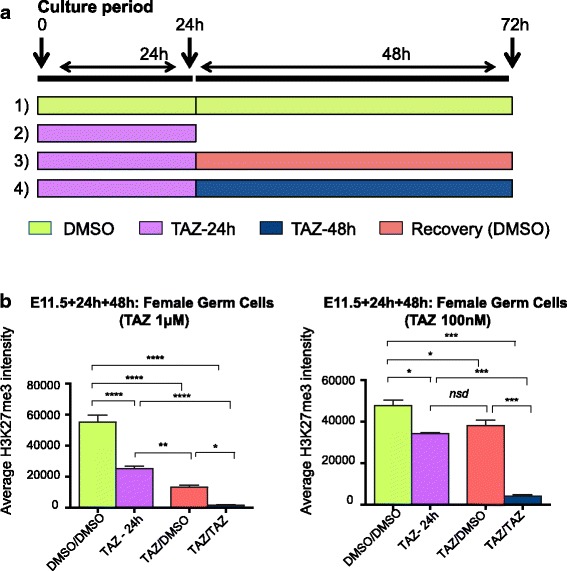
H3K27me3 was not recovered in oocytes after treatment of fetal ovaries with tazemetostat. Data summary from flow cytometric analyses of E11.5 XX gonads cultured for 24 h with DMSO (green), 100 nM or 1 μM tazemetostat (Blue), followed by drug removal and additional culture for 48 h in the presence of DMSO (green), 100 nM or 1 μM tazemetostat. Drugs were removed by washes with culture media after the initial 24 h of culture. Four treatment groups were used to collect control and experimental data. Treatment 1: DMSO/DMSO—24 h DMSO followed by 48 h DMSO (negative control). Treatment 2: TAZ—24 h tazemetostat (H3K27me3 depletion after 24 h drug treatment). Treatment 3: TAZ/DMSO—24 h tazemetostat followed by 48 h DMSO (H3K27me3 recovery after 48 h drug withdrawal). Treatment 4: TAZ/TAZ—24 h tazemetostat followed by 48 h tazemetostat (H3K27me3 depletion after 72 h drug treatment). **a** Summary of experimental pipeline for assessing H3K27me3 recovery after drug depletion in germ cells undergoing epigenetic reprogramming. **b** Average H3K27me3 staining intensity in germ cells for treatments 1–4 DMSO/DMSO, TAZ, TAZ/DMSO and TAZ/TAZ using 1 μM (left) and 100 nM (right) tazemetostat from cultured E11.5 XX female gonads (1 μM, *n* = 5 biological replicates, 100 nM *n* = 4 biological replicates). *P < 0.05, **P < 0.01, ***P < 0.001, ****P < 0.0001; one-way ANOVA plus post hoc Tukey’s multiple comparisons test. Error bars ± SEM

Collectively, these data demonstrate that tazemetostat potently depletes H3K27me3 in XX fetal germ cells as they undergo epigenetic reprogramming and entry into meiosis. However, all PRC2 components have been detected in fetal XX germ cells at E11.5 and E12.5 [14] indicating that germ cells may be able to recover H3K27me3 after drug exposure. To determine whether H3K27me3 could be re-established after depletion in fetal germ cells undergoing epigenetic reprogramming, we cultured E11.5 gonads for 24 h with DMSO, 100 nM tazemetostat or 1 μM tazemetostat; removed the drug by washing three times with drug-free culture media; and continued culture for an additional 48 h in the presence of 100 nM, 1 μM tazemetostat or DMSO (Fig. 5a). To ensure collection of control and experimental data at the end of the initial drug depletion period and after the recovery period, treatments were carried out on four treatment groups: (1) treatment with DMSO for 24 h followed by treatment with DMSO for 48 h, which provided a negative control to determine the H3K27me3 levels after 72 h culture without the drug; (2) treatment with tazemetostat for 24 h only, which determined the reduction of H3K27me3 after 24 h of treatment as a pre-recovery control; (3) treatment with tazemetostat for 24 h, followed by treatment with DMSO for 48 h, which determined the extent to which H3K27me3 recovered after tazemetostat exposure; and (4) treatment with tazemetostat for 24 h followed by treatment with tazemetostat for 48 h, providing a positive control to determine the H3K27me3 depletion after 72 h of drug treatment (Fig. 5a). Flow cytometry was used to determine the levels of H3K27me3 in the germ cells after each treatment. Germ cell H3K27me3 levels were depleted to a lesser extent at 100 nM, compared to 1 μM tazemetostat at 24 h, and these levels were maintained without restoration across the 48-h recovery period (Fig. 5b). At the higher dose (1 μM), germ cell H3K27me3 levels were depleted by 54% after 24 h and continued to decline over the 48-h recovery period (DMSO; *P* < 0.01, one-way ANOVA post hoc Tukey’s multiple comparisons test; Fig. 5b). Together, these data demonstrate that EZH2 inhibition rapidly, and potently, depleted germ cells of H3K27me3 and that recovery of H3K27me3 in fetal germ cells was dependent on tazemetostat dose.

### EZH2 inhibition potently depletes H3K27me3 in primary oocytes

**Fig. 6:**
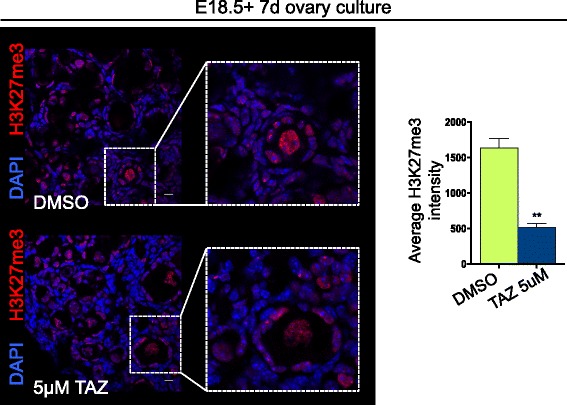
Tazemetostat depleted H3K27me3 in primary oocytes. Confocal images of H3K27me3 (red) and DAPI (blue) immunofluorescence in sections of E18.5 mouse ovaries cultured for 7 days with either DMSO (top panel) or 5 μM tazemetostat (bottom panel; 10 μm scale bars). Graphical representation of mean H3K27me3 staining intensity in DMSO treatment in green and tazemetostat treatment in blue, analysed by ROI manager, ImageJ. *P* < 0.005, Student’s *t* test; DMSO *n* = 4 and tazemetostat *n* = 3 biological replicates, three primary oocytes analysed per replicate. Error bars ± SEM

As tazemetostat is currently in clinical trials for treatment of cancer in patients aged 6 months to 18 years, it is possible that oocytes in the primordial follicle reserve will be affected by the drug in these patients. This is of particular relevance as the primordial follicle reserve provides all oocytes for the female reproductive life. To assess whether H3K27me3 was depleted in primary oocytes after birth, we cultured E18.5 mouse ovaries for 7 days with either vehicle control (DMSO) or 5 μM TAZ (Fig. 6). Primordial oocytes of E18.5 ovaries treated with 5 μM TAZ were significantly less enriched for H3K27me3 compared to controls (Fig. 6).

## Discussion

Drugs that target epigenetic modifying complexes hold great promise in oncology and potentially for treatment of a range of other diseases and disorders. These drugs are therefore likely to make a significant contribution to the treatment of human disease and provide substantial improvements in health outcomes. From this point of view, drugs targeting epigenetic modifiers are highly desirable and may be of significant benefit. However, these drugs also have the potential to affect epigenetic programming in the germline, with significant implications for health and developmental outcomes in offspring. There is therefore a need to assess the germline impacts of drugs that inhibit epigenetic modifying proteins to enable the informed clinical management of these drugs in patients of reproductive age.

In this study, we investigated the potential for drugs that inhibit EZH2 to alter H3K27me3 in growing oocytes, primary oocytes and the developing female germline. Significantly, tazemetostat prevented H3K27me3 enrichment in growing oocytes of adult females in vivo*,* and this effect was relatively stable as H3K27me3 did not recover after 10 days of drug withdrawal. In addition, EZH2 inhibition reduced H3K27me3 in the primary oocyte pool, indicating that the lifelong primordial follicle reserve is affected by these drugs. Finally, tazemetostat severely depleted H3K27me3 in developing oocytes undergoing epigenetic reprogramming and established that once depleted, H3K27me3 did not recover in these cells within the window analysed. Combined, these findings demonstrate that EZH2 inhibitors potently reduce H3K27me3 at all stages of female germline development.

Previous work, and our own experiments, using a mouse genetic model in which EZH2 was specifically deleted in growing oocytes demonstrated that maternal H3K27me3 was lost in the oocyte. Moreover, offspring had abnormal preimplantation development and were born with substantial growth restriction ([16], Prokopuk et al. unpublished data). Growth restriction is a risk factor for lifelong health impacts including metabolic disease [41–43]. Moreover, de novo germline mutations in human EZH2 result in Weaver syndrome, characterised by growth and skeletal defects and intellectual disability [44–46]. As these combined studies indicate that altered H3K27me3 in growing oocytes affects outcomes in offspring, it is likely that maternal exposure to EZH2 inhibitors and similar epigenetic modifying drugs will have the potential to impact on offspring growth and development. Although the specific impacts of EZH2 inhibitors on oocytes and offspring are yet to be determined, it is clear that much greater understanding of the potential germline impacts of these drugs in patients of reproductive age is required.

Understanding the impacts of drugs on both the parental germline epigenome and on offspring is of significant importance as a range of studies have shown that environmental impacts in the parent can affect outcomes in offspring through epigenetic mechanisms that are, by and large, poorly understood [47–59]. Moreover, although studies of other epigenetic modifying compounds, such as valproic acid, have demonstrated direct effects on the unborn fetus and on children of mothers treated during pregnancy [19], these studies have not addressed the potential intergenerational or transgenerational effects of drugs that target other epigenetic mechanisms in the germline.

In this study, tazemetostat caused severe depletion of H3K27me3 from growing oocytes after only 10 days of treatment and H3K27me3 had not recovered 10 days after treatment of the females had been terminated. This recovery period is equivalent to approximately half of the period required for a mouse oocyte to reach maturity from the primordial follicle stage [17]. The human oocyte takes approximately 350 days to grow and mature after recruitment from the primordial follicle pool [60, 61]. However, the current clinical guidelines recommend not to become pregnant for at least 30 days after treatment has been terminated. Consequently, after the termination of treatment, growing oocytes that were exposed to tazemetostat will remain for almost 11 months, and offspring conceived during this period may be also affected. Our findings in mice indicate that even after drug withdrawal there is a risk that H3K27me3 levels in oocytes will be affected for a significant period of oocyte growth and maturation. Based on oocyte growth and maturation periods of 21 days for mouse [17] and 350 days for human [60], we estimate that a similar withdrawal period in humans would require at least a full cycle of oocyte growth to allow the clearance of potentially affected oocytes. This is significantly longer than the current 30-day period recommended in most current tazemetostat clinical trials before patients should consider becoming pregnant. Therefore, it is possible that even after tazemetostat treatment has ceased in human patients, there will be a protracted period during which the oocyte epigenome will be affected and may have consequences for offspring.

Our data demonstrate that in growing oocytes, tazemetostat potently depleted H3K27me3 and H3K27me3 levels continued to decline until at least 10 days after treatment was withdrawn from the mice. Despite this, all core components of PRC2 were detected in growing oocytes, and H3K27me3 was enriched during oocyte growth demonstrating that PRC2 is normally active in growing oocytes. Similarly, H3K27me3 was not recovered in female fetal germ cells after tazemetostat treatment of fetal ovaries. We have previously shown that *Ezh2* is transcribed, EZH2 protein is present and EZH2 is required for redistributing H3K27me3 in XX germ cells during epigenetic reprogramming, demonstrating a functional role for EZH2 during this developmental period [14]. A possible explanation for the lack of H3K27me3 recovery after drug withdrawal is that once inhibited, EZH2 activity cannot recover, possibly because the drug irreversibly blocks and inhibits activity. This observation also implies that new EZH2 was either not synthesised in oocytes or the drug continued to block its activity throughout the withdrawal period. Retention of drug activity is unlikely as a previous study demonstrated an in vivo half-life for tazemetostat of only 4 h in mice [4], indicating that greater than 90% drug clearance in the first 24 h after drug withdrawal. Similarly, tazemetostat was washed out of the fetal ovarian cultures by changing the media for drug-free media and washing the wells multiple times. It therefore appears more likely that once blocked in either growing or fetal oocytes, EZH2 activity cannot readily recover. This may affect all stages of oocyte development and growth as EZH2 inhibition also compromised H3K27me3 in primary oocytes. It is therefore possible that tazemetostat and similar drugs may have persistent effects on all oocytes, including those in the primordial follicle pool. This is an important consideration when female children or patients of reproductive age are treated with the drug and is of direct relevance in current clinical trials involving patients aged 6 months to 18 years.

The current clinical guidelines clearly state that use of EZH2-inhibiting drugs is contra-indicated in patients during pregnancy. This position is presumably based on the direct impacts of EZH2 on somatic development in the fetus, but empirical evidence for this position is currently lacking. Recent reports of adverse impacts of the anti-epilepsy drug Epilim (valproic acid) [19] have highlighted the importance of a direct evidence-based position in developing stringent guidelines for these drugs in pregnancy. In addition to teratogenic impacts on somatic development in the fetus, in utero exposure to valproic acid has been associated with long-term effects on children, including decreased learning ability, autism and behavioural anomalies [19]. Similar outcomes in the fetus are likely for other drugs that inhibit epigenetic modifying complexes. However, it is also possible that drugs targeting epigenetic modifiers will cause intergenerational or transgenerational epigenetic effects in the patients’ children or grandchildren. Using gonad culture and in vivo treatments, respectively, we have clearly demonstrated that germline exposure to EZH2 inhibiting drugs severely affects epigenetic programming in the fetal and adult oocytes, highlighting the potent ability of these drugs to disrupt epigenetic programming in the germline and a need to further understand the impacts of these drugs on the germline.

Although new drugs that target epigenetic modifying enzymes offer important new therapeutic options for patients, it is clear that these drugs also have the capacity to significantly alter the germline epigenome and that these effects may persist for a significant period of time. One obvious way to avoid such effects in patients of reproductive age undergoing treatment with drugs that target epigenetic modifiers would be to employ germline preservation methods, such as sperm and oocyte freezing. However, as gamete preservation techniques are imperfect and not available to all patients, it is also clear that significantly more work is required to determine the molecular and phenotypic effects of EZH2 inhibitors in the germline and their potential to adversely affect subsequent children of female patients.

## Conclusions

Significantly, the potential impacts on the germline of drugs that target epigenetic modifying enzymes appear to be largely “off the radar” when the clinical safety of these drugs is being considered—most attention is reserved for the potential teratogenicity of the drugs rather than the potential germline impacts and subsequent effects on offspring. This study highlights an urgent need to determine risks of these and similar treatments for oogenesis and offspring outcomes.

## Methods

### Mouse strains, animal housing, breeding and ethics

Mice were housed at Monash Medical Centre Animal Facility using a 12-h light-dark cycle. Food and water were available ad libitum and room temperature was 21–23 °C with controlled humidity. Embryos were collected from *Oct4* [*Pou5f1*]-eGFP on 129T2svJ background males crossed with Swiss females. Females were checked for vaginal plugs daily and detection of a plug was noted as E0.5. Embryos E12.5 or older were sexed by the presence (male) or absence (female) of testis cords in gonads. E11.5 embryos were genetically sexed using PCR as described [62].

### Gonad collection and organ culture

All culture reagents were purchased from Life Technologies unless otherwise stated. Embryos were collected from Swiss females mated to 129T2svJ *Oct4*-eGFP transgenic males. Gonad plus mesonephros was cultured on 30 mm organotypic cell culture inserts (Merck Millipore; PICM03050) in 1200 μL culture media (250 μM sodium pyruvate, 15 mM Hepes, 1× non-essential amino acids) (Life Technologies, 11140), 1 mg/ml N-acetylcysteine (Sigma, A9165), 55 μM β-mercaptoethanol (Life Technologies, 21985) and 10% FCS in DMEM/F12 with Glutamax (Life Technologies, 10565) containing either DMSO (vehicle control) or tazemetostat (EPZ6438; SelleckChem, S7128). Culture media also contained 1× penicillin/streptomycin (Life Technologies, 15070). Gonads were randomly allocated to each culture treatment condition and cultured for 48 h in 37 °C/5% CO_2_ conditions. Culture media was refreshed daily. Gonads were processed for flow cytometry, FACS and immunofluorescence (IF).

### Flow cytometry

Gonad collection, dissociation, fixation, antibody staining and flow cytometry were performed as described [14, 40]. Gonad samples stained with rabbit IgG control antibody were used as negative controls to set flow cytometry gates for H3K27me3 intensity and mesonephros or limb samples were used as a germ cell negative control to gate for eGFP. Cell proliferation was assessed by dosing cultured gonads with 20 μM 5-ethynyl-2 deoxyuridine (EdU) for the final 2 hours of culture prior to tissue collection, dissociation and fixation. Cell cycle analysis was carried out as described [40, 63] with germ cells identified by their expression of mouse vasa homologue (MVH). Cells were stained with 20 μg/ml propidium iodide, allowing quantitation of cellular DNA content. Proliferation was measured by gating EdU-positive cells against propidium iodide to identify cells actively in S-phase. Cells in G1 and G2/M were identified by their DNA content and the absence of S-phase activity. All flow cytometry was performed on a FACS Canto instrument and data were analysed in FlowJo and Graphpad Prism. For all analyses, at least three biological replicates were analysed and statistical significance was determined using one-way ANOVA with Tukey’s multiple comparison or *t* test as appropriate. *P* values < 0.05 were considered significant.

### Tissue fixation and embedding

Gonads were fixed in 4% paraformaldehyde (PFA) in PBS for immunofluorescence (IF) overnight at 4 °C. Samples were washed twice in PBS and left in 30% sucrose in PBS overnight at 4 °C. Samples were then placed in disposable cryostat moulds (Sakura Finetek, 4565) filled with OCT (Sakura Finetek, 4583) and frozen in dry ice and stored at − 80 °C.

### Immunofluorescence

Immunofluorescence was carried out as previously described [14]. Eight micron sections were cut from OCT-embedded gonads fixed in 4% PFA, mounted on Superfrost Plus slides and dried for 5 min before immersing in 1× PBS. Sections were then permeabilized by incubation in 1% Triton X 100 (Sigma, T8787) in PBS for 10 min at room temperature (RT). Slides were washed three times for 5 min each in PBS. Sections were blocked in PBS containing 5% BSA (Sigma, A9647) and 10% donkey serum (Sigma, D9663) and incubated for 45 min at RT. Blocking solution was replaced by PBS containing 1% BSA and appropriately diluted primary antibodies (rabbit anti-H3K27me3 1:400, Cell Signaling Technology #C36B11; sheep anti-EED 1:100, R&D #AF5827; rabbit anti-EZH2 1:400, Cell Signaling Technology #D2C9; rabbit anti-SUZ12 1:100, Cell Signaling Technology #D39F6) and incubated for 1 h at RT. Slides were washed three times for 5 min in PBS and secondary antibodies diluted at 1:300 in 1% BSA in 1× PBS (Alexa Fluor, Life Technologies, Donkey anti Sheep 647 #A21447; Donkey anti Rabbit 594 #A21207). Secondary antibody incubation was carried out in a dark humidity chamber for 1 h at RT. Slides were washed three times in PBS (5 min each wash) and mounted in ProLong Gold**®** containing DAPI (Life Technologies, #P36931) and left in a dark box overnight to dry. For control slides, only a secondary antibody was applied. Confocal images were taken as single optical sections using a Nikon**®** C1 inverted confocal microscope. All pictures were taken at ×80, using a ×40 oil immersion lens. H3K27me3 mean intensity was measured in at least three biological replicates using ImageJ ROI manager to calculate the mean intensity for H3K27me3, and unpaired *t* tests were used to statistically analyse groups, with *P* ≤ 0.05 considered significant.

### In vivo injection of tazemetostat

Seven-week old C57Bl/6J females were subcutaneously injected with 220 mg/kg/day tazemetostat. Mice were weighed daily to ensure correct dosage. Dosage regime involved injecting mice once a day for 9 consecutive days and collecting females on the 10th day. Females were culled via cervical dislocation and ovaries were collected and processed for IF.

## Additional file

Additional file 1: **Figure S1.** Representative flow cytometric scatter plots demonstrating separation, gating and analysis of germ and somatic cells derived from gonads cultured with DMSO or tazemetostat. A. Representative flow cytometric plots of cells analysed from female cultured gonads. Oct4GFP-positive germ cells and Oct4GFP-negative somatic cells were separated and gated based on their respective positive or negative expression of Oct4GFP (left plot). Mean H3K27me3 staining intensity of individual germ (middle plot) and somatic cells (right plot) was quantified using FlowJo. B. Representative flow cytometric plots of cells analysed from limb control cells, which do not express Oct4GFP. This and similar controls facilitated the gating of Oct4GFP-positive and Oct4GFP-negative populations shown in A and B. C. Average H3K27me3 staining intensity in germ cells of E12.5 XX gonads cultured with DMSO, 1 μM and 5 μM tazemetostat for 72 h. **Figure S2.** Representative flow cytometric scatter plots demonstrating separation, gating and cell cycle analysis of germ and somatic cells derived from female E12.5 gonads cultured with DMSO or tazemetostat for 72 h. A. Oct4GFP positive germ cells and Oct4GFP negative somatic cells were separated and gated based on their respective positive or negative expression of Oct4GFP (left plots). The middle and right-hand plots represent cell cycle analyses based on incorporation of EdU and propidium iodide (PI) staining quantified using FlowJo (germ cell cycle-middle plots; somatic cell cycle-right plots). B. Average H3K27me3 staining intensity (left) and cell cycle state (right) in somatic cells from female E12.5 gonads cultured with DMSO or tazemetostat. ****P < 0.0001, one-way ANOVA plus post hoc Tukey’s multiple comparisons test; *n* = 3–5 biological replicates. (PDF 599 kb)

## Abbreviations

E: Embryonic day
EED: Embryonic ectoderm development
eGFP: Enhanced GFP
EPZ: EZH1/2 inhibitor, TAZ, tazemetostat/EPZ-6438
EZH1: Enhancer of Zeste 1
EZH2: Enhancer of Zeste 2
FACS: Fluorescence-activated cell sorting
GSK126: EZH1/2 inhibitor, GSK
H3K27me3: Trimethylated lysine 27 on histone 3
MVH: Mouse vasa homologue (also known as DDX4)
*Nsd*: No significant difference
PRC2: Polycomb repressive complex 2
SUZ12: Suppressor of Zeste 12
