# Supplementary material for: Pharmacological inhibition of EZH2 disrupts the female germline epigenome
Source: Clin Epigenetics. 2018 Mar 5;10:33. doi: 10.1186/s13148-018-0465-4 (PMC5836460; doi:10.1186/s13148-018-0465-4)

Supplementary Information for:

Pharmacological inhibition of EZH2 disrupts the female germline epigenome.

Lexie Prokopuk, Kirsten Hogg and Patrick S Western*

Centre for Genetic Diseases, Hudson Institute of Medical Research and Department of Molecular and Translational Science, Monash University, Clayton, Victoria, Australia 3168.

*Corresponding author:

Dr Patrick Western

Centre for Genetic Diseases,

Hudson Institute of Medical Research,

Clayton, Victoria, Australia 3168.

patrick.western@hudson.org.au

Tel: +61 3 8572 2673

Running title: Pharmacological alteration of the germline epigenome

Keywords: germline, oocyte, pharmacology, epigenetic, PRC2, H3K27me3, inheritance

Supplementary Figure 1. Representative flow cytometric scatter plots demonstrating separation, gating and analysis of germ and somatic cells derived from gonads cultured with DMSO or Tazemetostat. **A** Representative flow cytometric plots of cells analysed from female cultured gonads. Oct4GFP positive germ cells and Oct4GFP negative somatic cells were separated and gated based on their respective positive or negative expression of Oct4GFP (left plot). Mean H3K27me3 staining intensity of individual germ (middle plot) and somatic cells (right plot) was quantified using FlowJo. **B.** Representative flow cytometric plots of cells analysed from limb control cells, which do not express Oct4GFP. This and similar controls facilitated the gating of Oct4GFP positive and negative populations shown in A and B. **C.** Average H3K27me3 staining intensity in germ cells of E12.5 XX gonads cultured with DMSO, 1μM and 5μM Tazemetostat for 72h.

Supplementary Figure 1.

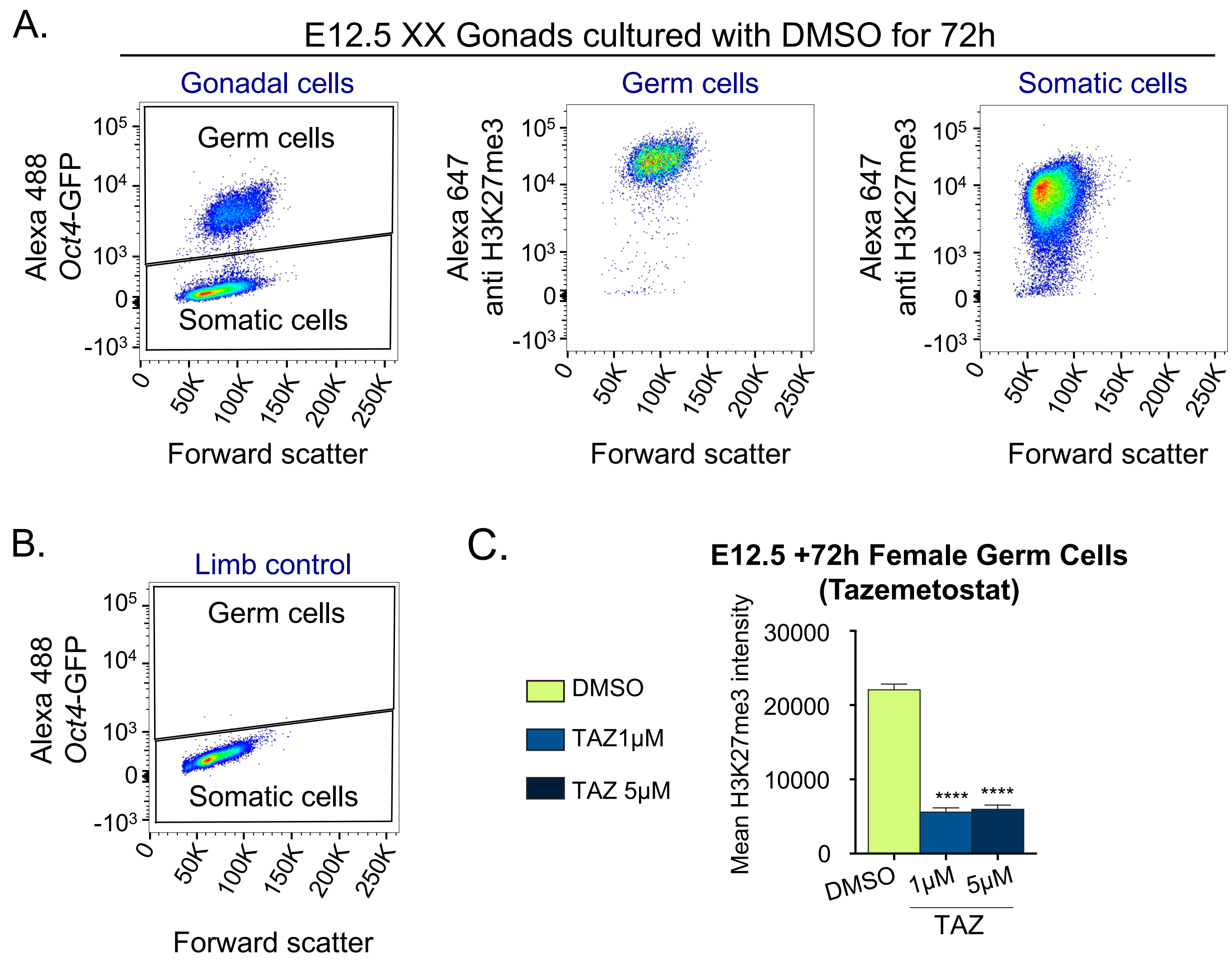

Supplementary Figure 2. Representative flow cytometric scatter plots demonstrating separation, gating and cell cycle analysis of germ and somatic cells derived from female E12.5 gonads cultured with DMSO or Tazemetostat for 72h. **A.** Oct4GFP positive germ cells and Oct4GFP negative somatic cells were separated and gated based on their respective positive or negative expression of Oct4GFP (left plots). The middle and right-hand plots represent cell cycle analyses based on incorporation of EdU and propidium iodide (PI) staining quantified using FlowJo (germ cell cycle- middle plots; somatic cell cycle- right plots). **B.** Average H3K27me3 staining intensity (left) and cell cycle state (right) in somatic cells from female E12.5 gonads cultured with DMSO or Tazemetostat. ****$P<0.0001$, one-way ANOVA plus post-hoc Tukey's multiple comparisons test; n=3-5 biological replicates.

Supplementary Figure 2.

A.

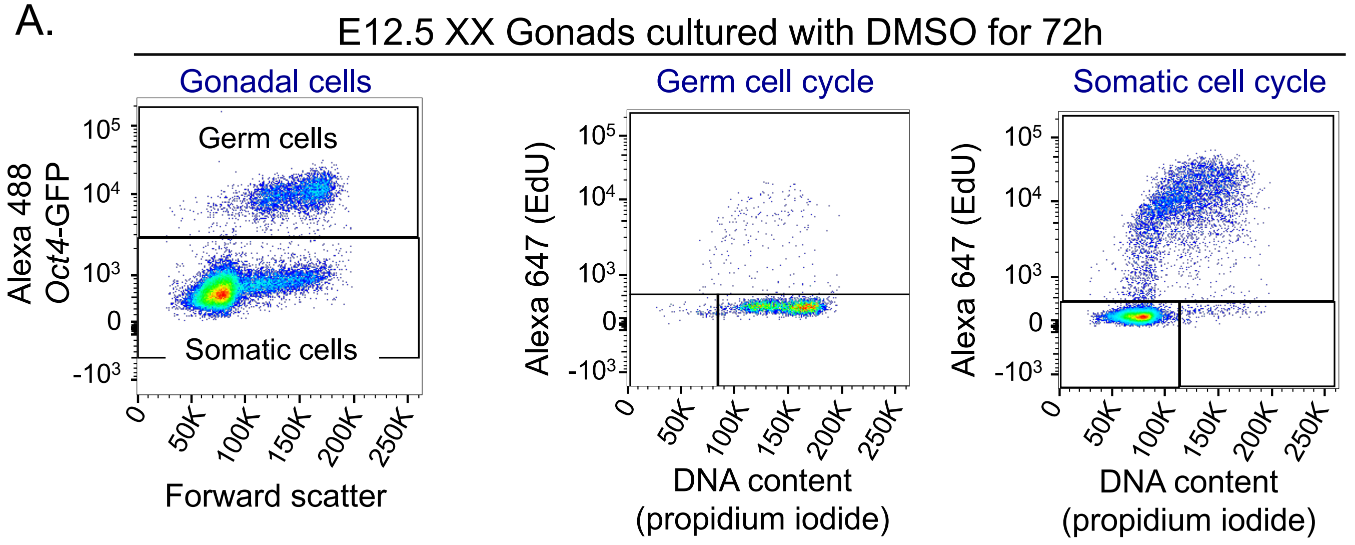

B.

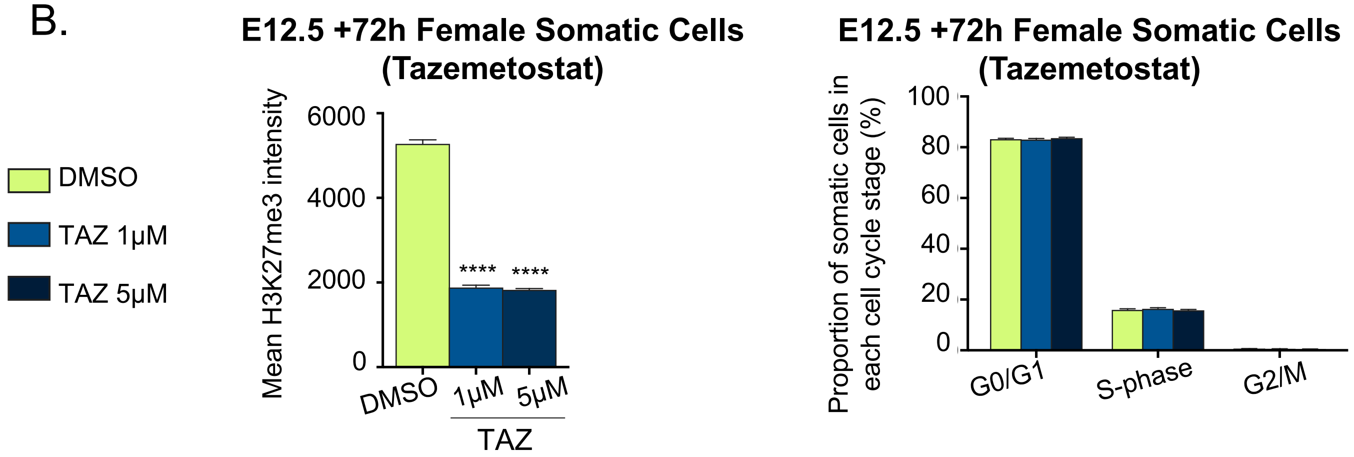

Supplement: Supplementary file 1 — Figure S1. Representative flow cytometric scatter plots demonstrating separation, gating and analysis of germ and somatic cells derived from gonads cultured with DMSO or tazemetostat. A. Representative flow cytometric plots of cells analysed from female cultured gonads. Oct4GFP-positive germ cells and Oct4GFP-negative somatic cells were separated and gated based on their respective positive or negative expression of Oct4GFP (left plot). Mean H3K27me3 staining intensity of individual germ (middle plot) and somatic cells (right plot) was quantified using FlowJo. B. Representative flow cytometric plots of cells analysed from limb control cells, which do not express Oct4GFP. This and similar controls facilitated the gating of Oct4GFP-positive and Oct4GFP-negative populations shown in A and B. C. Average H3K27me3 staining intensity in germ cells of E12.5 XX gonads cultured with DMSO, 1 μM and 5 μM tazemetostat for 72 h. Figure S2. Representative flow cytometric scatter plots demonstrating separation, gating and cell cycle analysis of germ and somatic cells derived from female E12.5 gonads cultured with DMSO or tazemetostat for 72 h. A. Oct4GFP positive germ cells and Oct4GFP negative somatic cells were separated and gated based on their respective positive or negative expression of Oct4GFP (left plots). The middle and right-hand plots represent cell cycle analyses based on incorporation of EdU and propidium iodide (PI) staining quantified using FlowJo (germ cell cycle-middle plots; somatic cell cycle-right plots). B. Average H3K27me3 staining intensity (left) and cell cycle state (right) in somatic cells from female E12.5 gonads cultured with DMSO or tazemetostat. ****P < 0.0001, one-way ANOVA plus post hoc Tukey’s multiple comparisons test; n = 3–5 biological replicates. (PDF 599 kb) [file 13148_2018_465_MOESM1_ESM.pdf]
